# Findings and Implications From an Evaluation of the Gold Star Campaign in Post-Ebola Guinea: The Role of Gender and Education

**DOI:** 10.9745/GHSP-D-21-00427

**Published:** 2022-02-28

**Authors:** Tilly Gurman, Darriel Harris, Sidikiba Sidibé

**Affiliations:** a Johns Hopkins Bloomberg School of Public Health, Center for Communication Programs Baltimore, MD, USA.; b Johns Hopkins Bloomberg School of Public Health, Baltimore, MD, USA.; c Johns Hopkins Center for Communication Programs-Guinea, Conakry, Guinea.

## Abstract

In post-Ebola Guinea, the Health Communication Capacity Collaborative (HC3) project worked to rebuild trust in the health system and increase the utilization of reproductive, maternal, neonatal, and child health services. Core to HC3 Guinea was the promotion of quality local health centers through the Gold Star campaign (2016–2017). The current study aimed to determine the relationship between campaign exposure and attitudes/intentions regarding local health centers, to inform communication efforts in future epidemics. Between June–July 2017, HC3 Guinea conducted a telephone survey with men (n=1,000) and women (n=2,000) aged 18–49 years. Multivariate regression analyses estimated the association between campaign exposure and attitudinal/intention outcomes for the overall sample as well as for gender- and education-stratified samples.

Survey results indicated that more than 30% of the sample recalled either the campaign (32.5%) or logo (37.6%). Statistically significant associations existed between exposure and various attitudinal outcomes. For example, regardless of gender/level of education, campaign recall was strongly associated with agreeing that the local health center improved in the last 6 months. Given that the campaign itself was short in duration, it is encouraging that attitudes changed with campaign exposure. Although a greater percentage of women than men held positive attitudes about health facilities, men seemed more influenced by campaign exposure.

The current study provides evidence that mass media campaigns can help rebuild trust in health care facilities after an epidemic. Study findings also stress the value of conducting stratified analyses by important demographic characteristics (e.g., gender, education). Stratified analyses can help identify meaningful differences and better tailor health promotion activities and achieve greater success. The recent recurrence of Ebola in Guinea has resulted in renewed discussions about ways to incorporate these evaluation findings into current programming, including exploring ways to address gender considerations in message design and overall program strategy.

## Abstract (precis)

During public health crises, such as an Ebola epidemic, people may lose trust in local health facilities. Short-duration mass media campaigns can improve attitudes about the quality of health facilities for men and women and can play an important role in encouraging future health service utilization.

## Teaser

**Key Findings:**

- Exposure to the Gold Star campaign was associated with a variety of attitudinal outcomes about perceived quality of health facilities for men and women in Guinea.
- The strongest association of exposure to the Gold Star campaign was on the attitude that the local state hospital or health center had improved in the last 6 months.
- There was a stronger association of exposure to the Gold Star campaign on attitudes among university-educated individuals compared to individuals with less than a university education.
- There was also a stronger association of exposure to the Gold Star campaign on attitudes among men compared to women.

**Key Implications:**

- Short-duration mass media campaigns, especially when coupled with improvements in the actual facilities and services, can help rebuild trust in and improve attitudes about local health care facilities after an epidemic. Being able to foster positive attitudes in a relatively short period of time can play an important role in increasing health service utilization after an epidemic.
- Because of men’s roles as both consumers of health services but also as gatekeepers to the consumption of health services by women and children in many settings, it is important to develop tailored campaign messages for men.

Résumé en français à la fin de l’article.

## INTRODUCTION

Between 2014–2016, Ebola wreaked havoc in Guinea. The epidemic disrupted and placed additional strain on an already fragile health system.^1^^,^^2^ Health services—especially reproductive, maternal, neonatal, and child health services (RMNCH)—as well as trust and confidence in health services, were already low among residents.^3^ Ebola’s rapid spread further fueled fear and distrust, with many people believing health workers themselves spread the disease.^4^^–^^6^ Increased fear and distrust resulted in decreased health care service utilization^7^ in a range of health areas such as HIV^8^ malaria testing and treatment,^9^ and RMNCH services.^10^^,^^11^ For example, 1 study found a 31% decrease in outpatient services, with child health services (e.g., vaccination, treatment for acute respiratory infection) most gravely affected.^10^ Similarly, a retrospective, observational cohort study of RMNCH service utilization in Beyla, Guéckédou, Kissidougou, Lola, Macenta, and N’Zérékoré districts found that post-Ebola, vaccination coverage for polio, measles, and yellow fever continued to decrease.^2^

As the country continued to recover from Ebola, the Health Communication Capacity Collaborative (HC3)—a 5-year global project funded by the United States Agency for International Development (USAID)—worked in Guinea’s hardest-hit communities to ameliorate these drops in health care service utilization. A primary objective of the overall project was to develop local capacities to design and implement health promotion programs for health, with a special focus on RMNCH services. In post-Ebola Guinea, HC3 Guinea worked with various partners—including the Guinea Ministry of Health—over approximately 18 months. Based on the observed trends in health care service utilization, HC3 Guinea focused on rebuilding trust in the health system, improving health care quality, and increasing the demand for RMNCH services.

HC3 Guinea implemented a 2-pronged approach that focused both on the services themselves as well as potential clients of services. The first component supported Guinea’s efforts toward the strengthening of the health system by rehabilitating health service facilities in regions severely impacted by the Ebola epidemic. The second component of HC3 Guinea’s efforts comprised the Gold Star (“Étoile d'or”) campaign and ran from May 2016 until May 2017. This campaign—originally started in 2012 and revitalized post-Ebola—aimed to build confidence in health services and increase utilization. A clinic that met specific quality criteria received accreditation, visible through a “gold star” which clinics prominently displayed inside and outside the facility. The Gold Star campaign branded its logo as a sign of high-quality services and marketed these facilities to the public via television and radio spots, billboards, promotional materials (e.g., t-shirts or stickers), as well as interpersonal/community channels (e.g., women’s networks, religious leaders, hygiene and health committee members, syndicates of motorcycle taxi drivers). The purpose of this study was to infer possible effects of campaign exposure by assessing whether exposure was associated with Guinean men’s and women’s attitudes toward clinics, as well as on their intention to utilize clinics in the future.

The Gold Star campaign aimed to build confidence in health services and increase utilization—giving accreditation to clinics that met specific quality criteria.

## METHODS

The current cross-sectional study assessed the association between exposure to the Gold Star campaign and attitudes about local health services as well as intention to access health services. GeoPoll, a firm that specializes in remote, mobile-based data collection, implemented a computer-assisted telephone interview (CATI) survey. The study included women (n=2,000) and men (n=1,000) aged 18–49 years in Guinea. Because HC3 Guinea was primarily interested in increasing demand for RMNCH services—which are most often accessed by women—the study, by design, purposefully sampled more women than men.

### Data Collection

The GeoPoll data collection team utilized randomized sampling to develop a database of mobile telephone numbers in Guinea, which data collectors used to identify and contact potential participants. GeoPoll accessed the full list of mobile phone subscribers of MTN—Guinea’s largest mobile phone provider—to randomly select a subsample of 135,000 mobile phone numbers. From this subsample, GeoPoll created random batches of 5,000 numbers from which data collectors would contact possible participants. GeoPoll would extract a new batch of 5,000 numbers once all numbers had been called in that batch. In total, GeoPoll extracted 3 batches of 5,000 numbers until it reached the desired sample size of 2,000 women and 1,000 men.

Data collection occurred during June–July 2017, with each CATI lasting about 25 minutes. To offset the burden of the phone, study participants received an airtime credit equivalent to US$0.50. To achieve the desired study sample size, data collectors contacted approximately 12,570 phone numbers across all regions of Guinea, with 5,896 individuals not answering the call (46.9%), 1,103 individuals not eligible (8.5%), and 2,571 individuals ending the call early (20.5%). The total response rate for the survey was 23.9%, which is considered high for phone surveys.

### Human Subjects Considerations

Data collectors obtained oral consent from potential participants before commencing the survey. The current study received ethical approval from both the Johns Hopkins University Bloomberg School of Public Health Institutional Review Board (IRB#6807) and the Comité National d'Ethique pour la Recherche en Santé (National Ethics Committee for Research in Health) in Guinea (IRB#036/CNERS/17).

### Variables

The survey included a range of questions about demographics (e.g., education, radio ownership, and television ownership), exposure to the Gold Star campaign, various attitudes about the local/state health facility, and intention to access health care services. Due to the 12-month duration of the campaign and the fact that certain behaviors would likely not change within that short time frame, the study focused on measuring shifts in attitudes and intention.

Due to the fact that certain behaviors would likely not change within the 12-month campaign duration, the study focused on measuring shifts in attitudes and intention.

Two variables, generated from separate survey questions about Gold Star campaign recall, measured exposure. The first variable captured general campaign recall, based on participants’ response to a question about whether, in the last month, they had seen or heard information about improving the quality of services in health facilities. The second variable measured aided recall to the Gold Star logo, based on a question about whether they had ever seen a logo that includes a gold star around an image of a health care provider.

The current study explored the association between each measure of exposure and 3 separate outcomes.

1. The first outcome was a composite measure of discrete attitudes about the perceived quality of health facilities. Participants agreed or disagreed with the following statements:
  - The local state hospital or health center is clean.
  - I am confident that the local state hospital or health center will keep people safe and free from infection.
  - If I were to attend the local state hospital or health center, I would receive a warm welcome from the health provider.
  - If I were to attend the local state hospital or health center, I trust the health provider would meet my health care needs.
2. The second outcome measured participants’ attitude about perceived improvement of their local health facility in the last 6 months.
3. The third outcome in this study assessed an individual’s intention to go to a health provider the next time he/she needs health care.

### Data Analysis

Researchers used Stata version 16.1 (StataCorp, LLC) to analyze the quantitative survey data. Factor analysis— using principal component analysis followed by promax rotation—helped identify the variables to include in the composite measure of attitudes regarding perceived quality (α=0.74). The scores for this composite ranged from 0 to 1, with 1 indicating agreement with all 4 positive attitudes about health facilities. Chi-square tests of differences in proportions explored differences by gender regarding exposure to Gold Star and outcomes and assessed statistical significance of those differences. Multivariate logistic regression measured the association between the 2 measures of campaign exposure and the 3 outcomes of interest. Multivariate regression models with the full study sample controlled for age, gender, level of education (less than university compared to university or higher), and language of survey administration, regardless of statistical significance. To more clearly infer differences in possible campaign effects by gender and education level, researchers decided to also conduct separate stratified regression models. The gender- and education-stratified multivariate regression models controlled for language of survey administration and age. The stratified models compared the associations between exposure and outcomes by gender or level of education.

## RESULTS

### Participant Characteristics

The study included a total of 3,000 participants (women=2,000; men=1,000) (Table 1). The majority of respondents (74.2%) were aged 18–29 years. Although more than half of surveyed respondents lived in Conakry, the sample included respondents from all of Guinea’s other 7 regions and 33 prefectures. In terms of the highest level of education completed, 47.8% of participants reported completing university. Most participants reported living in households owning radios (72.3%) and televisions (81.7%).

**TABLE 1.** Characteristics of Respondents to Telephone Survey on Gold Star Campaign, 2017, Guinea (N=3,000)

**Characteristic**	**No. (%)**
Gender	
Men	1,000 (33.3%)
Women	2,000 (66.6%)
Age range, years	
18–29	2,227 (74.2%)
30–39	544 (18.3%)
40–49	229 (7.6%)
Language of survey	
French	2,599 (86.6%)
Other (Pullar, Soussou, or Malinké)	401 (13.4%)
Highest level of education completed	
None	406 (13.5%)
Elementary	81 (2.7%)
Junior High	337 (11.2%)
Senior High	733 (24.4%)
University	1,434 (47.8%)
Household owns radio	2,170 (72.3%)
Household owns television	2,451 (81.7%)
Recalled Gold Star campaign	976 (32.5%)
Recalled Gold Star logo	1,129 (37.6%)

The overwhelming majority of survey respondents expressed positive attitudes about the local state hospital/health center. For example, 86.6% of respondents agreed that if they were to attend the local state hospital/health center, health care providers could meet their health care needs. Similarly, 83.5% of respondents agreed that their local state hospital/health center was clean and 82.4% agreed that their local state hospital or health center will keep people safe and free from infection. In addition, 75.4% of respondents perceived that the local state hospital or health center had improved over the last 6 months. When comparing men and women, a greater percentage of women held positive attitudes about health care services (Table 2). For example, 77.1% of women compared to 72.1% of men agreed that the local health center has improved in the past 6 months (*P*<.05).

**TABLE 2.** Survey Respondents Differences in Exposure to the Gold Star Campaign, Attitudes About Health Services, and Intention to Access Services in Guinea, by Gender (N=3,000)

	**Males** **No. (%)**	**Females** **No. (%)**	**Overall** **No. (%)**
Recalls campaign^a^	352 (35.2%)	624 (31.2%)	976 (32.5%)
Recalls logo	386 (38.6%)	743 (37.2%)	1,129 (37.6%)
Agrees that the local state hospital or health center is clean^a^	797 (79.7%)	1,707 (85.4%)	2,504 (83.5%)
Agrees that the local state hospital or health center will keep people safe and free from infection^a^	786 (78.6%)	1,685 (84.3%)	2,471 (82.4%)
Agrees that if at a health center, he/she would receive a warm welcome from health provider^a^	798 (79.8%)	1,686 (84.3%)	2,484 (82.8%)
Agrees that if at a health center, provider would meet his/her needs	851 (85.1%)	1,748 (87.4%)	2,599 (86.6%)
Agrees that health center has improved in the past 6 months^a^	721 (72.1%)	1,541 (77.1%)	2262 (75.4%)
Intends to go to a health provider the next time needs health care	956 (95.6%)	1,925 (96.3%)	2,881 (96.3%)

^a^Statistically significant differences (*P*<.05) between males and females.

More than 75% of respondents perceived that the local state hospital or health center had improved over the last 6 months.

Among survey respondents, 32.5% reported having seen or heard information about improving the quality of services in health facilities in the last month (Table 2). Among these respondents, the most common message recalled was, “They are improving facilities to help Guinea be healthy.” A slightly higher percentage of men versus women recalled having heard about quality services (35.2% versus 31.2%, respectively; *P*<.05). In addition, 37.6% of respondents overall reported recall of the Gold Star logo. The difference in logo recall between men and women was not statistically significant. Among respondents who reported seeing the logo, the most common meaning associated with the logo was quality health care facilities.

### Multivariate Analysis Results

When looking at results from the multivariate regression models (Table 3 and Table 4), 4 patterns appear. (Each table presents results from all stratified and unstratified multivariate regression models for 3 different outcomes, for a total of 27 models per table.) First, exposure to the campaign was associated with a variety of outcomes. In the 6 multivariate regression models for the total sample—which controlled for gender, level of education, television ownership, radio ownership, language of interview, and age—2 models for general campaign recall and 1 model for logo recall achieved statistical significance. When stratifying by education, the associations were stronger for individuals with university education compared to those with less education. Of the 3 stratified models run for general campaign recall limited to those with university education, 2 achieved statistical significance, compared to only 1 model for individuals with less than university education. Similarly, of the 3 stratified models run for logo recall limited to those with university education, 2 achieved statistical significance, compared to only 1 model for individuals with less than university education.

**TABLE 3.** Association^a^ Between Survey Respondent’s General Gold Star Campaign Recall and Attitudes About Health Facilities/Intention to Access Health Facilities in Guinea, by Gender and Level of Education

	**Men (n=1,000)**			**Women (n=2,000)**			**Total Sample (N=3,000)**		
	**Overall**	**University**	**<University**	**Overall**	**University**	**<University**	**Overall**	**University**	**<University**
Perceived quality of the local health facility	1.33^b^	1.58^b^	1.08	1.24^b^	1.43^b^	1.07	1.28^b^	1.50^b^	1.07
*P* Value	.041	.015	.719	.05	.021	.645	.005	.001	.592
Agrees that the local health facility has improved in last 6 months	2.55^b^	2.46^b^	2.76^b^	1.94^b^	2.23^b^	1.69^b^	2.17^b^	2.33^b^	2.03^b^
*P* Value	<.001	<.001	<.001	<.001	<.001	<.001	<.001	<.001	<.001
Intends to go to health provider the next time health care needed	1.05	1.52	.712	0.85	.74	.91	0.90	.99	.83
*P* Value	.895	.389	.492	.518	.477	.768	.594	.983	.481

^a^ Each cell represents a separate regression model. All models controlled for television ownership, radio ownership, language of interview, and age. The models for the total sample also controlled for gender and education.

^b^ Results indicate statistically significant findings (*P*<.05).

**TABLE 4.** Association^a^ Between Gold Star Logo Recall and Attitudes About Health Facilities/Intention to Access Health Facilities in Guinea, by Gender and Level of Education

	**Men (n=1,000)**			**Women (n=2,000)**			**Total Sample (N=3,000)**		
	**Overall**	**University**	**<University**	**Overall**	**University**	**<University**	**Overall**	**University**	**<University**
Perceived quality of the local health facility	1.57^b^	1.87^b^	1.30	0.95	1.08	.86	1.14	1.34^b^	.98
*P* Value	.001	.001	.208	.634	.582	.285	.103	.011	.864
Agrees that local health facility has improved in last 6 months	2.45^b^	2.90^b^	2.05^b^	1.25^b^	1.26	1.27	1.60^b^	1.72^b^	1.50^b^
*P* Value	<.001	<.001	.002	.049	.168	.137	<.001	<.001	.002
Intends to go to health provider the next time health care needed	3.03^b^	2.42	4.41	0.98	1.46	.783	1.37	1.79	1.12
*P* Value	.009	.093	.051	.929	.390	.417	.134	.082	.679

^a^ Each cell represents a separate regression model. All models controlled for television ownership, radio ownership, language of interview, and age. The models for the total sample also controlled for gender and education.

^b^ Results indicate statistically significant findings (*P*<.05).

Second, the influence of the campaign seemed strongest for the attitude that the local state hospital or health center had improved in the last 6 months. Statistically significant associations (*P*<.05) for agreeing with this attitude were overwhelmingly present for both types of exposure, in the nonstratified as well as stratified analyses. For both general and campaign recall, the strongest association for the entire sample was with agreeing that the local health center/state hospital had improved in the last 6 months (General recall: OR=2.17, *P*<.001; Logo recall: OR=1.60, *P*<.001). For general campaign recall, the association was even stronger when stratified by level of education, with those with university education having a stronger association with this attitude compared to those with less than university education (OR=2.33, *P*<.001 versus OR=2.03, *P*<.001, respectively). For general campaign recall, the strongest association with the attitude that the local state hospital or health center had improved in the last 6 months occurred for men with less than university education (OR=2.76, *P*<.001). For logo recall, the strongest association with the attitude that the local state hospital or health center had improved in the last 6 months occurred for men with university education (OR=2.90, *P*<.001).

The influence of the campaign seemed strongest for the attitude that the local state hospital or health center had improved in the last 6 months.

Third, exposure to the general campaign as well as to the logo seemed to have influenced men more so than women. For general campaign recall, 5 of the 9 total multivariate regression models for men achieved statistical significance compared to only 4 of 9 models for women. Similarly, for logo recall, 6 of the 9 total multivariate regression models for men achieved statistical significance, compared to only 1 of 9 models for women.

Finally, intention to go to a health provider the next time health care is needed had the least number of statistically significant associations with general campaign exposure. No association existed for the entire sample or in any of the stratified analyses for general campaign recall. For logo recall, the only statistically significant association existed for men overall (OR=3.03; *P*=.009). Interestingly, this association was the strongest across both types of exposure and all outcomes, for both the entire sample as well as the samples stratified by gender and level of education.

## DISCUSSION

HC3 Guinea implemented the 12-month Gold Star campaign as a central component of its larger portfolio aimed at restoring trust in and increasing utilization of RMNCH services at improved local health facilities. The current study identified novel findings regarding the association between Gold Star campaign exposure—measured through both general campaign recall and logo recall—and favorable attitudes about health facilities for men and women in Guinea. Even though the final study sample skewed toward urban and highly educated individuals, it was representative of mobile telephone owners in Guinea given its robust sample size. Overall findings indicate that for study participants—all of whom had access to mobile phones and 47.8% of whom were university-educated—exposure to the Gold Star campaign was associated with favorable attitudes about the quality of local health services. In addition, the study found that these associations were stronger for men compared to women.

The current study provides promising evidence that the short-running Gold Star campaign improved attitudes regarding quality health services and offers implications for future programs and research. First, exposure to the Gold Star campaign was associated with a variety of attitudinal outcomes across the entire sample. Given that the campaign itself was relatively short in duration, it is encouraging to see that favorable attitudes were associated with campaign exposure, both via general campaign recall as well as recall of the actual Gold Star logo. These findings suggest that the campaign might have been an important part of the effort to improve health care utilization, particularly when coupled with continual improvements in the physical appearance of health service facilities, the interpersonal interactions with health care providers and other health facility staff, and the overall quality of the actual services provided. Although the current study does not directly explore health service utilization, Gold Star facility data documented a trend in increased utilization after the Gold Star Campaign compared to the same time period from the previous year.^12^

These findings suggest that the campaign might have been an important part of the effort to improve health care utilization.

Second, study findings point to the ability of the campaign to influence male and female study participants’ perceptions that the local state hospital or health center had improved in the last 6 months. This association occurred for both general campaign recall as well as logo recall. Considering that the campaign coincided with rehabilitation efforts of the health facilities but produced statistically significant differences in attitudes for general campaign and logo recall, study findings suggest that the campaign may have had an additive effect unaccounted for by rehabilitation alone.

Third, although a greater percentage of female than male study participants held positive attitudes about health facilities (Table 2), males seemed more influenced by exposure to the Gold Star campaign. It could be that pre-campaign attitudes of the local health facilities may have been more negative among male study participants than female study participants, since they may be less likely to have direct experience with the services themselves. Study findings suggest that campaigns like the Gold Star campaign may have a greater capacity to positively influence perceptions among men. Moreover, these findings may speak to the fact that women may also be more likely to visit the local health facilities for RMNCH services and have direct experience with the improved services themselves. Therefore, their positive attitudes may reflect their lived experience and not exposure to the campaign itself. This finding suggests that the lived experiences to the actual changes in the local state health clinics may supersede campaign messaging and logo branding.

The current study complements other literature about the importance of integrating a gender lens when developing, implementing, and evaluating global health programs during and after epidemics and pandemics.^13^^–^^16^ The recent coronavirus disease (COVID-19) global pandemic has emphasized the reality that during pandemics women are disproportionately impacted by health service disruption and related health outcomes.^13^ Similarly, during the 2015–2016 Zika pandemic women experienced deficits in routine health care access because of a lack of autonomy over health care decision making.^17^ Moreover, during the 2014–2016 Ebola outbreak, more women died from childbirth complications than from the disease itself.^17^ With these insights in mind, the differences observed in this study’s findings from the gender-stratified analyses stress the importance of tailoring messages specifically to men that are grounded in the local context.^15^ Even though women tend to utilize RMNCH services more than men, men may play a key gatekeeper role by providing financial or emotional support.^16^ Therefore, by changing men’s attitudes, health communication campaigns could indirectly help to increase women’s future access to health care services.^14^^,^^16^ In particular, tailoring messages to men that highlight aspects of quality and safety may inspire them to welcome the utilization of routine RMNCH services by their female partner. The study also reinforces the value of conducting disaggregated analyses by gender, to better understand the role of gender when evaluating global health programs.^13^^–^^15^

This study complements other literature about the importance of integrating a gender lens when developing, implementing, and evaluating global health programs during and after epidemics and pandemics.

Finally, although intention was not associated with campaign recall and only associated with logo recall for male study participants, it is important to note that approximately 96% of both male and female study participants intended to go to a health provider the next time they need health care. Therefore, although exposure to the campaign was not associated with intention, the fact that almost all participants held the intention suggests that future campaigns may want to capitalize on that almost-universal intention by encouraging people to act on their intention. Future formative research may want to further explore intention and try to uncover the factors that influence individuals to move from intention to action to better craft messages and programs aimed at motivating people to act.

### Limitations

As with any research study, the current study experienced several limitations. First, the study was cross-sectional in nature, limiting the ability to have comparison groups by level of exposure. The study used 2 measures of self-reported exposure to compare individuals, which is not as strong a study design as one in which there is a true comparison group. At the same time, comparing individuals who reported exposure to individuals without offers some insight into the possibility of campaign exposure to influence attitudes.

Second, the study was not representative of the national population of Guinea, likely due to the CATI methodology via mobile phones. The decision to conduct a mobile phone survey was based on financial and time constraints of implementing a face-to-face household survey. Even before data collection began, the study team recognized that mobile phone users may skew toward more highly educated and urban populations. Yet in 2017, Guinea had approximately 6 million mobile phone subscribers.^19^^,^^20^ In addition, between 2007 and 2017, the annual growth rate of mobile phone subscribers in Guinea was 17%.^20^ These trends indicating a growing market in Guinea supported the decision to conduct the survey using CATI. The approach to sample from the mobile subscriber database of the country’s largest mobile operator hoped to generate a more representative sample.

In the end, however, the final study sample skewed more urban and educated. With 47.8% of the sample having a university degree, the socioeconomic status of study participants was likely similarly higher than the general population in Guinea. In the 2018 Demographic Health Survey (DHS), in contrast, only 8% of women and 18% of men were university educated.^21^ At the same time, the 2018 DHS also indicates that, regardless of residence or socioeconomic status, the majority (68.1%) of individuals in Guinea who sought health care in the previous month did so at a public sector facility.^21^ In urban areas that percentage was 55.5% and among individuals in the fourth and fifth quintile of socioeconomic status, those percentages were 62.0% and 52.9%, respectively.^21^ For women, the primary audience of the Gold Star campaign, these percentages increase to 61% in urban areas, and in the fourth and fifth quintile of socioeconomic status, those percentages were 66.8% and 60.3%, respectively.^21^ Even with the study’s limitation on generalizability, inferences about exposure to the Gold Star campaign possibly influencing attitudes toward the local health center in a post-Ebola context remain valuable.

Third, a variable asking for a respondent’s total number of children encountered data quality concerns. The lead researcher noticed nonsensical responses to the question and confirmed that several data collectors had misinterpreted the question when conducting the phone survey. As a result, the lead researcher removed this data from further analysis. This variable would have provided the opportunity to control for the number of children respondents have, a characteristic that may influence an individual’s receptivity to novel information about RMNCH services.

## CONCLUSION

Even given its limitations, this study remains insightful in light of the ongoing COVID-19 global pandemic as well as the resurgence of Ebola in Guinea. During and since the 2014–2016 Ebola epidemic in Guinea, international attention has prioritized health system strengthening and resulted in multiple actors partnering with the Guinean government.^1^^,^^18^ Without increasing utilization of general health services, however, improvements in health systems will fall flat and fail to achieve lasting behavior change among the population. Health communication campaigns, such as Gold Star, are critical programmatic complements to health systems strengthening activities.^11^ Study findings demonstrated that mistrust in health care facilities and health care providers, even when exacerbated by a public health crisis, can be overcome through a thoughtful short-run health communication campaign that highlights confidence in revitalized local facilities and services. Therefore, as the Guinean government strives to ensure a resilient health system during COVID and the resurgence of Ebola, health communication campaigns can serve to concurrently reinforce trust and encourage health service utilization. The Guinea-based program implementation team for current activities related to COVID-19 and Ebola have revisited these study findings and started working on incorporating these insights into recent programming. The key implications highlighted above may also be useful to other similar programs in other settings, both in the current worldwide COVID-19 pandemic as well as in future global health crises.

## Translation

**En Français**

**Résultats et Implications d'une Évaluation de la Campagne Gold Star (Etoile d’OR) en Guinée dans la période Post-Ebola: Le Rôle du Genre et de l'Éducation**

**Résumé:** En Guinée, après Ebola, le projet Health Communication Capacity Collaborative (HC3) est intervenu pour rétablir la confiance dans le système de santé et accroitre l'utilisation des services de santé reproductive, maternelle, néonatale et infantile. HC3 Guinée a mis l’accent sur la promotion de la des centres de santé locaux de qualité à travers la campagne D’Etoile d’Or (2016-2017). La présente étude visait à déterminer la relation entre l'exposition à la campagne et les attitudes/intentions concernant les centres de santé locaux, mais aussi informer sur les efforts de communication pouvant servir lors des futures épidémies. Entre juin et juillet 2017, HC3 Guinée a mené une enquête téléphonique des hommes (n=1 000) et des femmes (n=2 000) âgés de 18 à 49 ans. Des analyses de régression multivariées ont permis d'estimer l'association entre l'exposition à la campagne et des résultats en matière d'attitude/intention pour l'échantillon total ainsi que pour les échantillons stratifiés par genre et par niveau d'éducation.

Les résultats de l'enquête ont indiqué que plus de 30% de l'échantillon se rappelaient de la campagne (32,5%) ou du logo (37,6%). Des associations statistiquement significatives existaient entre l'exposition et divers résultats des attitudes. Par exemple, indépendamment du genre et du niveau d'éducation, le rappel de la campagne était fortement associé au fait d'être d'accord avec l'idée que le centre de santé local avait amélioré pendant les 6 derniers mois. Étant donné que la campagne elle-même était de courte durée, il est encourageant de noter que les attitudes ont changé avec l'exposition à la campagne. Bien qu'un pourcentage plus élevé de femmes que d'hommes ait eu des attitudes positives à l'égard des centres de santé, les hommes ont semblé plus influencés par l'exposition à la campagne.

La présente étude fournit des données indiquant que les campagnes médiatiques peuvent contribuer à rétablir la confiance dans les centres de santé après une épidémie. Les résultats de l'étude soulignent également le mérite de mener des analyses stratifiées en fonction de certaines caractéristiques démographiques importantes (par exemple, le genre, l'éducation). Les analyses stratifiées peuvent aider à identifier des différences notables, à mieux adapter les activités de promotion de la santé et à obtenir de meilleurs résultats. La récente résurgence d'Ebola en Guinée a donné lieu à de nouvelles discussions sur les moyens d'intégrer les résultats de ces évaluations dans les programmes actuels, notamment en explorant les moyens de prendre en compte les considérations de genre dans la conception des messages et la stratégie du programme.

**Peer Reviewed**
